# Comparison of Mask-Wearing Behavior on Social Media and Its Relationship With Demographic Characteristics During the COVID-19 Pandemic: Social Media Data Analysis Between the United States and Japan

**DOI:** 10.2196/78236

**Published:** 2026-04-08

**Authors:** Kiki Ferawati, Wan Jou She, Shoko Wakamiya, Eiji Aramaki

**Affiliations:** 1 Graduate School of Science and Technology, Nara Institute of Science and Technology, 8916-5 Takayama-cho, Ikoma, 630-0192, Japan, 81 743-72-5250; 2 Faculty of Mathematics and Natural Sciences, Sebelas Maret University, Surakarta, Indonesia; 3 Faculty of Information and Human Sciences, Kyoto Institute of Technology, Kyoto, Japan

**Keywords:** COVID-19, mask-wearing, cultural differences, classification, map, correlation

## Abstract

**Background:**

Social media is one of the most accessible and extensive sources of data for tracking and understanding public reactions to COVID-19 policies. Cultural differences between the United States and Japan have resulted in highly distinctive policies and public reactions in each country.

**Objective:**

This study aims to analyze the public opinions surrounding COVID-19 mask mandate through 1,102,876 and 560,873 geo-tagged tweets from the United States and Japan during the period from 2020 to 2022. We conducted 3 stages of analysis—relevance to COVID-19 masks, stance for or against masking, and whether the tweets indicate users wearing masks—to understand individuals’ stance towards the mask mandate and their actual mask-wearing behavior.

**Methods:**

We adopted a semisupervised approach to enhance BERT (Bidirectional Encoder Representations from Transformers) classification results due to data imbalance, which were then visualized through time series and map representations.

**Results:**

In the United States, our data showed that individuals with a bachelor’s degree or higher, as well as those living in states with higher household incomes, are positively correlated with positive attitudes toward mask-wearing. In contrast, in Japan, those with higher education levels or individuals aged 65 years and older were positively correlated with tweets categorized as having a stance against the mask mandate. Key events in Japan, such as the announcement of the state of emergency and the Olympics, served as major triggers for the number boost in public opinion.

**Conclusions:**

Our analysis of over 1.6 million tweets from the United States and Japan revealed that public opinion shifted notably in response to major events and policy changes during the COVID-19 pandemic. While some trends align with previous research, correlations with education, age, and income suggest that social media data may reflect underlying societal divisions and algorithm-driven biases.

## Introduction

Countries around the world have adopted a diverse strategy for managing the COVID-19 pandemic, as summarized in the government responses index in 2020 when COVID-19 first impacted the globe [1]. The implementation of different types of policies by countries is influenced by various factors, including demographics and geographical considerations. Among the measures taken to curb the spread of the disease, the mask mandate is recognized as one of the most widely adopted policies in several countries. Early in the pandemic, the Centers for Disease Control and Prevention (CDC) advised people in the United States to wear masks as a preventive measure [2], which was also adopted in a few other countries [3]. While the Japanese government did not mandate mask-wearing, its population had already been accustomed to wearing masks even before the COVID-19 outbreak, during the pandemic, and after [4].

The comparison between the United States and Japan is a frequently studied topic among researchers due to cultural contrasts and variations in Western and Eastern societal conventions [5,6]. Cultural views also contributed to how people in the 2 countries reacted differently as a response to health policies during the pandemic [7]. During the COVID-19 outbreak, both countries displayed contrasting attitudes regarding policy creation to control the spread of the disease and adhere to mask mandates. Undoubtedly, the vast number of social media posts is often viewed as a valuable resource for comprehending, analyzing, and even informing policymakers about societal perceptions or attitudes toward an occurrence. However, a significant obstacle for cross-cultural comparison studies is handling data from multilingual social media data. From the process of data labeling, classification, and processing, the differences between the characteristics of the 2 languages might provide interesting results to be analyzed.

Social media serves as a cost-effective and accessible alternative for assessing public responses in addition to surveys [8]. Changes in people’s behavior during pandemics can be observed on social media platforms like X (formerly Twitter), as demonstrated during the 2009 H1N1 pandemic [8,9] and the COVID-19 pandemic [10]. X reflects real-time insights into individuals’ reactions [11,12], making it a convenient yet highly sensitive tool for assessing the risk associated with events or interventions that may have downstream effects on policy-making and implementation [13,14]. Social media might also show declining sentiment on topics related to COVID-19 restrictions throughout the pandemic, as described in the results of a study using tweets in the 3 cities in the United States [15]. Other than previously mentioned studies, the content from social media posts, such as tweets, can also be used to assess possible outbreaks by using tools such as large language models [16].

The debate surrounding masking has generated one of the most widely discussed topics throughout the pandemic, which could be clearly traced and documented on X posts [17]. Previous studies using location information in X have been conducted, such as to predict the prevalence of ZIKV cases in the United States by filtering tweets [18]. The studies used a bounding box as the boundary of the observed area, excluding tweets from Canada and Mexico. Spatial mapping shows similar patterns observed across the United States. Another study also uses geotagged tweet data, covering discussions about the #MeToo movement in the United States [19]. The results show further discourse about the movement, including the descriptions of abuse often related to the case. These existing studies suggested that geotagged tweets might provide information about ongoing social media discussions in a certain area.

This study aims to compare the discussion about masking in COVID-19 between the United States and Japan on social media and its changes throughout the pandemic stages. In the COVID-19 period, the 2 countries have different approaches to the response to the pandemic, resulting in different policies and rules. This difference was also reflected in how people responded, which can also be seen on social media. Mask-wearing, as one of the policies during COVID-19, generates a lot of discussion among the public. Some are supporting, and some are against mask-wearing. Despite their small number, people who did not want to wear masks are a vocal minority in sharing their opinion [20]. Due to all those differing opinions, while mask-wearing is indeed an important effort to mitigate the spread of COVID-19, there are still many debates about the necessity of masks [14,21]. A geographical focus in the analysis was also interesting to explore, as a survey showed that mask-wearing as a public health behavior might exhibit spatial heterogeneity, suggesting a different approach in the area based on country-level analysis in the United States [22].

In this study, we discussed the public opinions surrounding the COVID-19 mask mandate through geo-tagged tweets from the United States and Japan during the period from 2020 to 2022 in 3 stages of analysis: starting from its relevance to COVID-19 masks, stance for or against masking, and whether the tweets indicate users wearing masks. By discussing the factors and correlation of the observed tweets, we aim to understand individuals’ stance towards the mask mandate and their actual mask-wearing behavior.

## Methods

### Ethical Considerations

This study did not require participants to be involved in any physical or mental intervention. This research also did not use personally identifiable information, thus exempted from institutional review board approval in accordance with the Ethical Guidelines for Medical and Health Research Involving Human Subjects stipulated by the Japanese national government.

### Data

The data in this study consists of geo-tagged tweets collected between January 1, 2020, and December 31, 2022, using Academic Research Access (discontinued in mid-2023). This dataset comprised 1,102,876 English tweets and 560,873 Japanese tweets. Note that we used the same dataset as our previous study on multilanguage data annotation [23]. Examples of paraphrased tweets showing their opinion about mask-wearing are shown in Textbox 1.

Textbox 1. **Example of tweets in English and Japanese.**

English:

- Against: Wearing this mask at work is exhausting. I’m sweating like crazy even from the smallest tasks.
- Not against: Unexpected perk of wearing a mask: it keeps your face nice and warm in freezing weather! \#MaskUp \#StayWarm \#WinterHack

Japanese:

- Against: 普段あまりこういうことは言わないようにしてるけど, どうしてまだみんな外でマスクをしてるの？室内でも特に必要とは思えないし, もう外してもいいんじゃないかな, 本当に。 (I usually try not to say things like this, but why is everyone still wearing masks outside? I don’t really see the need for them indoors either. Isn’t it okay to stop wearing them already? Honestly.)
- Not against: それに, マスクをしていない人の割合もかなり高め。ちょっと気が緩みすぎてない？ (Besides, the number of people not wearing masks is pretty high. Aren’t people getting a little too careless?)

Demographic data were accessed from the Portal Site of Official Statistics of Japan [24] and Census Bureau data of the United States [25], obtained from the 2020 census. Previous studies have indicated that demographic characteristics such as sex, ethnicity, age, marital status, and employment status affect participation in preventive behaviors in the United States [26]. In this study, we focused on several demographics for population characteristics at the state/prefecture level: total population, population by age, education level, information on foreign members/race, and income (Table 1). For Japan, the education level was obtained from the number of employed persons aged 15 years and older.

**Table 1.** Demographics variable used in correlation calculation.

Demographics	Variable
Population^a^	Total population Population of working age^b^ Population of elderly (older than 65 years)
Education level^c^	Population completed bachelor or higher
Income	Household income
Race information	Race index (the United States), Number of foreign residents (Japan)

a Total population from 2020 census.

b Percentage of people aged 18‐64 years (the United States) and 15‐64 years (Japan).

c Percentage of people completed bachelor or higher from population aged older than 25 years (the United States) from American Community Survey (2021) and population of employed person aged older than 15 years (Japan) from 2020 census.

### Preprocessing of Tweets

The first preprocessing step involved validating each tweet’s location tag. Given the various types of location information in geo-tagged tweets, we focused on city-level information found in the “full-name” entity of the tweets. Instances where the geo-tagged location did not match the city lists in the United States and Japan were removed [27,28]. Next, we applied basic preprocessing steps, including changing usernames to common handles, addressing links, converting emojis to text, and removing duplicates and NAs.

Finally, we ensured that the tweets contained the keyword “mask” for English tweets and “マスク” (mask in Japanese) for Japanese tweets. In some cases, these keywords appeared as usernames or links, providing insufficient information or relevance to the topics and were thus eliminated in the process.

### Data Annotation

The annotation for our 1100 pairs of tweets was done by 4 annotators, 2 for each English and Japanese. We used Cohen kappa as the measure to evaluate interannotator agreement between annotators [29] and interpret the agreement results [30]. The complete detailed approach and evaluation are explained in Ferawati et al [23]. To combine the annotated label between the annotators, we assigned the agreed label as the final label and assigned the disagreed label as not relevant, not against, and unknown for each stage.

### Classifier Training

X studies often use human annotators with the aid of machine learning classifiers [31,32]. We designed a multilingual guideline to obtain a suitable data annotation for this study [23]. The results of the annotation show a case of imbalanced data, with one category having a much smaller number of tweets compared with another. However, due to the time and resources required for human annotation, it is not practical to annotate a large number of tweets. To address this issue, we propose a semisupervised approach by using the BERT (Bidirectional Encoder Representations from Transformers) score from BERT-based classification method as additional data for training. By including additional training sets from predicted tweets, we hope to improve the performance of the classifier to solve the cases, especially in public perception and attitudes for COVID-19 masking in the 2 countries.

An existing study explained that BERT surpassed traditional machine learning algorithms, such as Logistic Regression, Support Vector Machine, Random Forest, and Naive Bayes, where BERT was supported by its ability to understand contextual information appearing in social media [33]. BERT was also widely used to analyze and derive conclusions. Based on the results of these studies, we decided to use BERT-based models for our study. We trained the annotated datasets using BERT-based classifiers, RoBERTa base model [34] for English tweets and BERT base Japanese [35] for Japanese tweets. To evaluate the models, we set aside 15% of the annotated data as the test set and applied 5-fold cross-validation to the remaining data. The final model was selected from the fold with the best results on the test set, evaluated using *F*_1_-macro to address data imbalance.

Due to the limited number of annotated data, we included all 1100 annotated tweets. For each language, tweets annotated with the same categories by both annotators were assigned to those categories. In cases of conflicting annotations—for example, where annotator 1 marked a tweet as not relevant while annotator 2 marked it as relevant—we categorized the tweet as not relevant. The same approach was applied for the stance stage (categorized as unclear, then assigned to not against) and mask-wearing (categorized as unknown).

### Semisupervised Approach

We observed an imbalance in the categories of our annotated data, which likely contributed to the low performance of our models. Due to constraints in obtaining additional annotated data, we implemented a semisupervised approach for the stance and mask-wearing stages of the tweets. The semisupervised approach was a common measure taken to obtain more annotated data without a significant increase in annotation costs [36]. In this study, we stored the BERT outputs from the predictions and filtered them based on determined thresholds for each category. We considered 2 methods of adding pseudo-labeled data: adding an equal number of tweets to both categories and adding tweets only to the minority category. Using softmax-transformed values of the BERT output for each class, we selected a set of data to be added to the training. The objective for implementing the approach is to obtain labels with a higher confidence from the obtained softmax-transformer values as an additional sample for training. We maintained the chosen split of training and validation from the previous step in classifier training.

The selected tweets from this step were then included as additional training data using the same parameters and model as in the previous subsection. The data for validation and testing remained the same as the baseline. We experimented with several combinations of thresholds for each class in each stage (against and not for stance; no, yes, and unknown for mask-wearing), while endeavoring to keep the number of selected additional tweets balanced. When determining the threshold value for the selection, we selected a certain value to balance our objectives: retaining sufficient confident predictions for the reliability of our pseudo-label and having enough additional samples to meaningfully expand our dataset. The threshold was set by observing the transformed BERTScore from the prediction results, plotting them into a histogram with the aid of descriptive statistics of the score to determine the appropriate threshold for filtering. We then compared the results of each training.

### Correlation

We calculated the correlation between the predicted percentages and demographic data from the 2 countries, the United States and Japan, using Pearson correlation. In this study, we considered 3 levels of significance for the *P* values with a significance level of .05, .01, and .001.

## Results

### Interannotator Agreement

For the annotation phase, we used only a subset of the entire dataset. Considering the reasonable workload of our annotators, we selected 1100 tweets from each language, as reported in another publication focusing on the annotation of a multicultural dataset [23]. Although the agreement levels for stance and mask-wearing stage are still less than <0.60—a score that was categorized as moderate by Cohen (1960) [29] but inadequate by McHugh (2012) [30]—they are deemed acceptable for our study (Table 2).

**Table 2.** Cohen kappa for the annotation results. The percentage in each round shows the number of samples annotated. The agreement is calculated for each round and each part of the samples.

Stage	Language	
	English	Japanese
1: Relevancy	0.79	0.92
2a: Stance	0.46	0.59
2b: Mask-wearing	0.55	0.47

The number of tweets varies substantially across the country, with certain states or prefectures having far more tweets than others. The difference in number is primarily observed in densely populated areas, such as the capital or popular destinations. Among tweets identified as relevant to COVID-19, California leads the United States in mask-related discussions, while Tokyo tops the list in Japan. A list showing the top and bottom tweet counts for states (in the United States) and prefectures (in Japan) is provided (Table 3).

**Table 3.** Top and bottom 3 of tweet counts in the United States and Japan.

Ranking	United States		Japan	
	State	Tweet count	Prefecture	Tweet count
Top 3				
	California	113,148	Tokyo	85,836
	Texas	75,517	Kanagawa	27,737
	New York	60,317	Osaka	26,725
Bottom 3				
	Vermont	1314	Kochi	1197
	North Dakota	1079	Tottori	1149
	Wyoming	776	Shimane	1049

### Classifier Training

We fine-tuned roberta-base and bert-base-japanese-v3 with our annotated tweets to predict the rest of the data, a method commonly used for social media data [37,38]. We trained the classifier using this annotated data and then used it to predict the remaining tweets. The parameters for our final model used the AdamW optimizer, with a batch size of 32, 10 epochs with early stopping callback, learning rate of 0.00002, and weight decay of 0.01.

We used the entire sample of 1100 tweets for relevancy classification (Table 4). In the first stage of relevancy classification, we achieved the highest *F*_1_-macro scores for both English and Japanese texts compared with the majority baseline. However, the stance and mask-wearing stage did not yield similar results, showing only a marginal increase from the majority baseline. This indicates that the models are still unable to correctly predict the minority categories. These results also mirror the annotation results, where annotators struggled to achieve higher interannotator agreement.

**Table 4.** Results of BERT (Bidirectional Encoder Representations from Transformers)-based classifier. Only tweets annotated as relevant by both annotators are included as a sample for Stage 2a: Stance and Stage 2b: Mask-wearing. As the category is heavily imbalanced for all the stages, we calculated the majority baseline by assigning majority classes as the predicted category in each stage: relevant for relevancy, not against for stance, and unknown for mask-wearing.

Stage	Language	Number of samples, n	Majority baseline	*F*_1_-macro
1: Relevancy				
	English	1100	0.44	0.78
	Japanese	1100	0.39	0.75
2a: Stance				
	English	880	0.47	0.61
	Japanese	706	0.45	0.69
2b: Mask-wearing				
	English	880	0.31	0.31
	Japanese	706	0.29	0.40

### Semisupervised Approach and the Final Classifier

By adopting a semisupervised approach to train our classifier, we were able to improve second-stage *F*_1_-scores that were nearly as high as those from the first stage—0.68 for English tweets and 0.72 for Japanese tweets.

Based on the results for the stance stage, the chosen threshold with a more balanced number of tweets in each category (eg, an additional 650 in the “against” category, bringing the total to 755 “against” and 775 “not against” for English data) achieved a better *F*_1_-macro score than other alternatives.

When it comes to classifying mask-wearing behavior, maintaining a balanced sample for re-training is challenging due to the imbalance of labels in the annotated data. In English tweets, only 46 tweets were predicted as “not wearing mask” out of the entire dataset, so we included all the predicted tweets in the model. The best result was an *F*_1_-score of 0.487, a substantial increase from the previous score of 0.301 with the original annotated data. For Japanese tweets, our best model only yielded an *F*_1_-score of 0.48.

For the mask-wearing stage, the distribution of BERT score outputs was much more skewed than for stance in both languages. Because it was challenging to maintain balanced numbers for each category, we experimented with various threshold combinations to assess performance. Due to the difficulty in balancing the numbers, the improvement in the model performance seems rather limited. Therefore, it should be considered the best our classifiers could achieve and is acceptable to be used in our following tasks.

The final classifier model was trained on a combined dataset of the original annotated data and the semisupervised data. The remaining tweets were classified using the final model. We deemed the *F*_1_ -score from the previous semisupervised results acceptable for our study and used it as a basis for generating a map of stance toward the mask mandate and actual mask-wearing behavior from related tweets in the United States and Japan, using both English and Japanese tweets.

### Time Series Observation of the Classification Results

**Figure 1.**
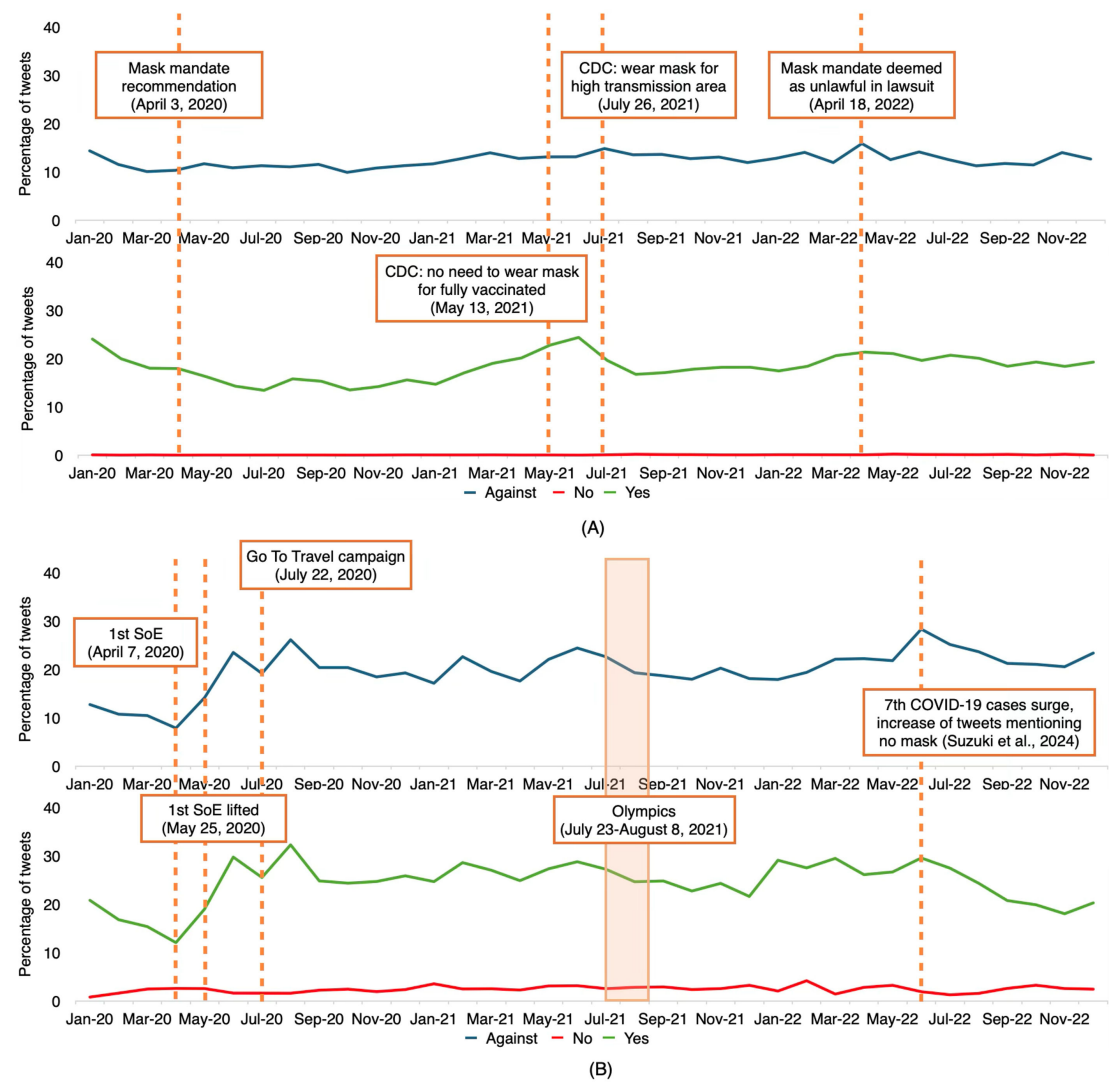
Time series plot of tweets against masking in (**A**) the United States and (**B**) Japan. Some events and policies happening throughout the period are highlighted in the figure. The upper part of the graph shows the percentage of tweets against mask-wearing, while the lower part shows tweets mentioning users wearing a mask (green) and not wearing a mask (red) [39]. CDC: Centers for Disease Control and Prevention; SoE: state of emergency.

The tweets were collected over a 3-year period, capturing the trend from early, peak, to post-peak periods of the COVID-19 pandemic. Throughout this time, various events and policies occurred, potentially influencing tweet volume and content (Figure 1). In the figure, no observable trend in the stance against COVID-19 masking is evident in the United States, suggesting little change in people’s opinion about mask-wearing from early 2020 to the end of 2022. Both countries show a decreasing trend in tweets against masking from January 2020 to April, followed by an upward trend after relevant departments released recommendations about mask-wearing.

Throughout the observed period, the CDC released several guidelines on the utilization of masks as a preventive measure. They initially mandated the use of masks in April 2020 and later revised the recommendation after the development of vaccines, such as lifting the recommendation to wear masks for fully vaccinated individuals in May 2021. In the month following CDC’s revision of their mask-wearing guidance to no need to wear a mask if vaccinated, tweets classified as “not wearing mask” reached their lowest point, while tweets classified as “wearing mask” peaked (Figure 1). Subsequently, the CDC released another recommendation to wear masks in high-transmission areas in July 2021, which is reflected in interesting changes observed in the time series plot of mask-wearing stages. The number of tweets indicating “not wearing mask” substantially increased from 0.03% in June 2021 to 0.21% in August 2021, while tweets about “wearing mask” saw a decrease from 24.41% in June 2021 to 16.76% in August 2021. In April 2022, as a result of a lawsuit regarding mask mandates deemed unlawful in Florida, nationwide mask mandates for airplanes and public transportation were no longer enforced [40]. As shown in Figure 1A, in the same month, a peak was observed in the stance against mask mandates in the United States, and tweets classified as “not wearing mask” peaked a month after the ruling, observed in May 2022.

In Japan, there are 3 noticeable fluctuations in the figure, closely related to COVID-19 policies. We observed some significant changes when the government announced a state of emergency (SoE) in April 2020 and subsequently lifted it in May 2020. After a dip in the percentage of tweets expressing a stance against masking after the first SoE in April 2020, the first peak appeared a month after the first SoE was lifted. These fluctuations are also consistent with findings from another study on responses to COVID-19 waves in Japan [41]. Another study discussed that there are indeed signs of disruptions due to COVID-19 in X, which shows top concerns during the SoE period and some impacts of the pandemic on societies in Japan [42]. The second peak appeared at the beginning of the Olympics. The Tokyo Olympics, held from July 23 to August 8, 2021, also influenced the observed changes in the figure. Before the Olympics began, in June 2021, there was a slight increase in tweets expressing a stance against mask-wearing, as shown in Figure 1B. The third peak appeared in June 2022, coinciding with the 7th surge in COVID-19 cases and an increase in “no mask” tweets, as observed by Suzuki et al [39].

### Map Comparison

#### Tweets Against Masking

The final classifications from the selected model were presented in the form of maps, depicting the percentage of tweets classified as against mask-wearing in the United States (at the state level) and Japan (at the prefecture level). Table 3 reveals a significant discrepancy in tweet frequencies between states and prefectures. For instance, California has over 100,000 relevant tweets, while other states like Wyoming have fewer than a thousand tweets. Similarly, in Japan, Tokyo, the capital, has the highest number of relevant tweets, with over 85,000, while Tottori has the fewest tweets, with approximately 1000.

**Figure 2.**
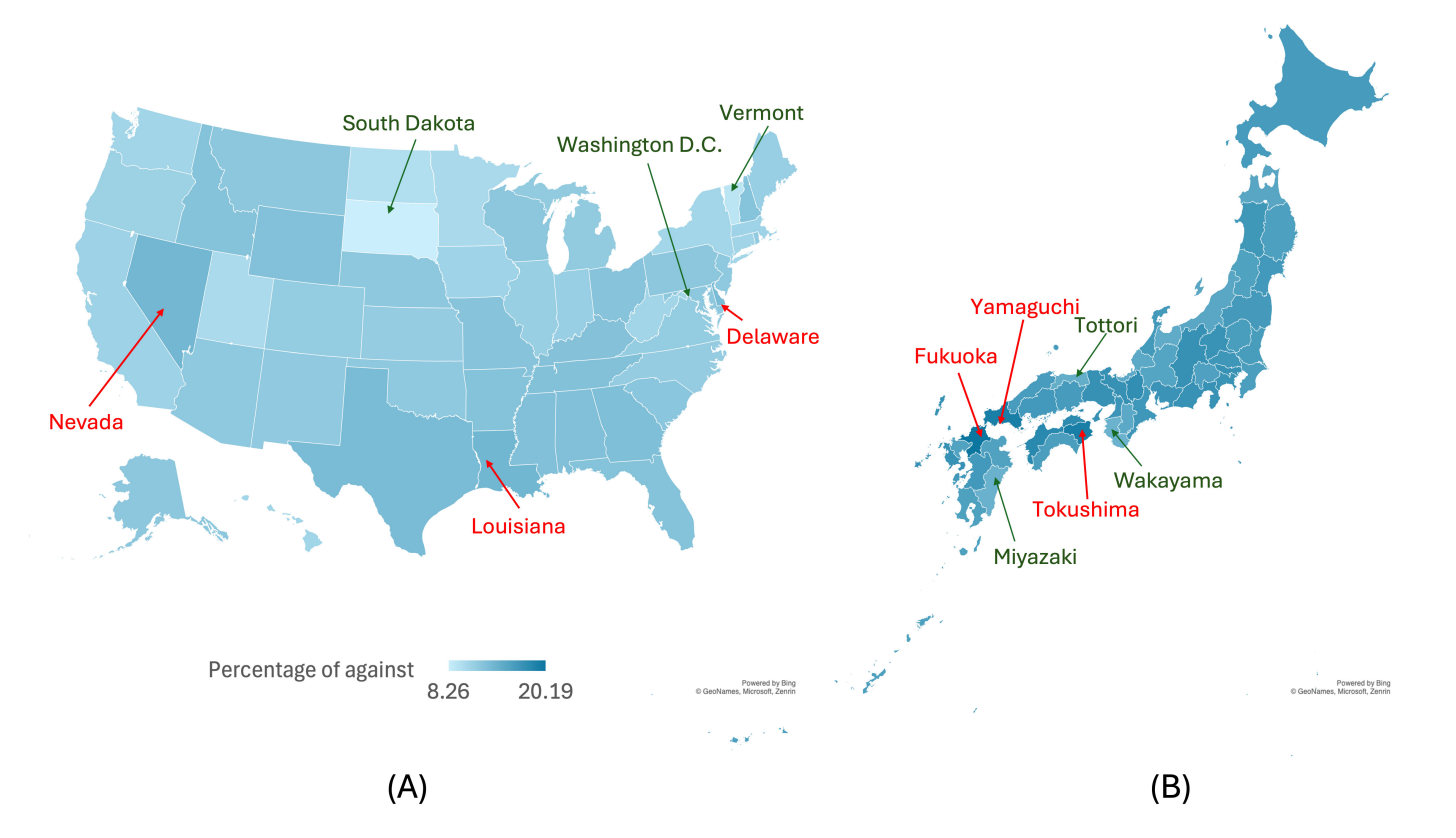
Percentage of tweets against masking in (**A**) the United States and (**B**) Japan. The same color scale is used for both the United States and Japan. The top 3 prefectures/states with the highest percentage are marked in red text and arrow, while the bottom 3 are marked in green text and arrow.

In the United States, Louisiana, Nevada, and Delaware were the 3 states with the highest percentage of tweets against masking, ranging from 13.28% to 13.75% (Figure 2). The 3 states with the lowest percentage are Washington, DC; Vermont; and South Dakota, with about 8%‐9%. On the other hand, in Japan, the percentage of tweets against masking is observed to be higher, ranging from 19.28% to 20.19% in Fukuoka, Yamaguchi, and Tokushima prefectures, with the lowest percentage observed at about 14% in Tottori, Miyazaki, and Wakayama prefectures.

#### Mask-Wearing Classification Results

**Figure 3.**
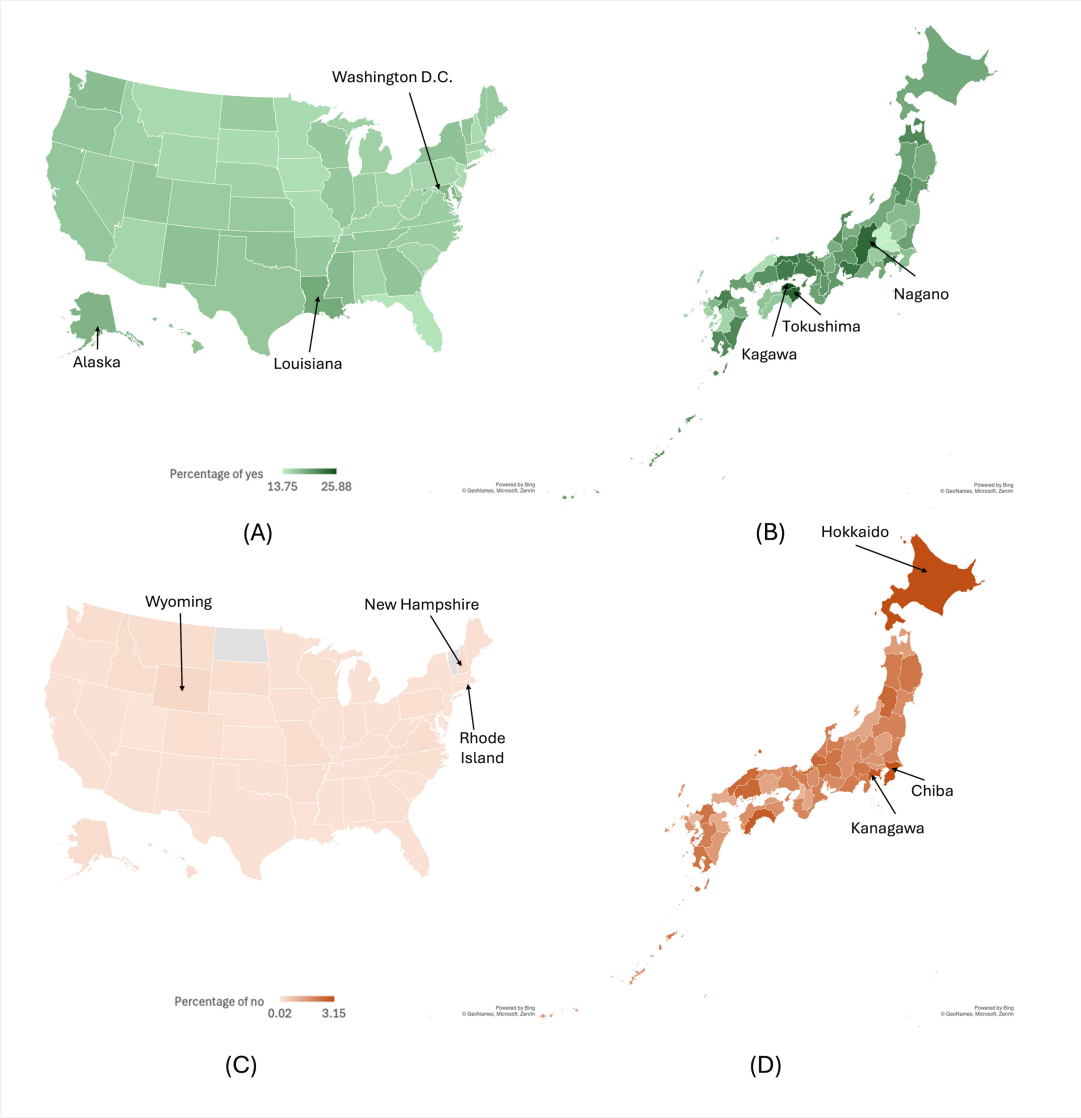
Percentage of tweets classified as wearing a mask in (**A**) the United States and (**B**) Japan, and tweets classified as not wearing a mask in (**C**) the United States and (**D**) Japan. The state/prefecture name shown denotes the top 3 areas with the highest percentage. The same color scale is used for both the United States and Japan. Gray shows no predicted data for the area.

The analysis and maps for mask-wearing behavior tweets were divided into 2 categories: those classified as “not wearing” and “wearing” masks (Figure 3). Since some states or prefectures have a much smaller number of tweets, the count of tweets classified as “not wearing” is reduced accordingly. For English tweets, the total count of “not wearing” tweets was very small, at 574, resulting in no tweets classified in the category in several states and causing the percentage to appear as 0. Wyoming has the highest percentage of tweets classified as “not wearing,” at 0.26% (2 out of 776). In contrast, Louisiana leads in the percentage of “wearing” mask tweets, at 19.12% (2122 out of 11,100); followed by Washington, DC (1638 out of 8845, 18.52%); and Alaska with about 18.33% (333 out of 1817). However, a careful interpretation is necessary for these figures, as the number of tweets was relatively small, especially for the tweets mentioning not wearing masks. The tweets were much smaller in the behavioral aspects of mask-wearing, so the resulting maps might not have enough data for a state-level conclusion.

On the Japan side, there were 8462 tweets classified as “not wearing” masks, a higher number compared with the United States results. However, a big portion of these tweets was still concentrated in Tokyo, with over 2000 tweets, while the other 31 prefectures had fewer than 100 tweets each. In terms of the percentage of tweets classified as “not wearing” masks, Hokkaido has the highest percentage at 3.15%. For tweets classified as “wearing” masks, Kagawa has the highest percentage at 25.87%.

### Correlation With Demographics Data

We calculated the correlation between stance percentages and the demographic data from the country census for both the United States and Japan (Table 5). In the United States, we observed a negative correlation of −0.384 between household income and stance against masking percentages. Although this correlation is relatively low, it is still significant within the 1% level of significance threshold. This suggests that states with higher incomes tend to have lower percentages of tweets against masking. Additionally, education level also shows a significant negative correlation, showing that with an increase in the population completing higher studies, there was an observed decrease in tweets with a negative stance towards the mask mandate. In Japan, we found that the number of people older than 65 years showed a positive correlation with the percentage of tweets against the mask mandate at the prefecture level. Similar results were observed in the number of people who completed a bachelor or higher degree.

**Table 5.** Correlation calculation of tweets percentage with the demographics data from the United States and Japan. The table only displayed a significant correlation.

Variable	Correlation	95% CI	*P* values
The United States			
Population completed bachelor and higher	−0.456	−0.653 to −0.200	.001
Household income	−0.353	−0.577 to −0.080	.013
Japan			
Population of elderly (older than 65 years)	0.295	0.009 to 0.537	.044
Population completed bachelor and higher	0.310	0.024 to 0.548	.034

## Discussion

### Principal Results

#### Key Events That Drove the Surge in COVID-19–Related Tweets

The time series plot illustrates the changes in stance against masking and mask-wearing tweets in the United States and Japan. The major fluctuations in the data points often coincide with governments announcing mask-related policies or major public events such as lockdown or the Olympics during the COVID-19 period. Such a phenomenon suggested that individuals tend to be prompted to express themselves on social media by major movements from government or public events, as previous studies also indicated [43-46]. In addition to the temporal trend, the geographical visualizations from the final results provide a general landscape of public responses to the mask-related policies divided by the municipal regions on social media (referred to as “X” in this study) in the United States and Japan. Information regarding the percentage of tweets against masking is displayed for each state or prefecture (Figure 3). Interestingly, Washington, DC, emerges as the state with the lowest proportion of tweets against masking and the highest percentage of tweets mentioning mask-wearing. This suggests that the state exhibits less resistance to mask-wearing and actively follows mask guidance.

Based on the correlation results between tweets and demographic data, education level and household income were found to be significantly correlated in the United States. Specifically, the population of people who have completed a bachelor’s degree and higher is negatively correlated with a stance against masking. This result indicates that with the increase in the higher education levels of the population in the states, we observed a decrease in the number of tweets against masking. This observation aligns with findings from a previous study based on a survey in the United States, which reported that individuals with at least a college degree are less likely to refrain from wearing face masks [47]. Additionally, the same study found that households with lower incomes were more likely to exhibit the same behavior of not wearing masks, an insight consistent with our findings.

Previous studies indicate that the behavior of older and younger people changes throughout the pandemic, with older individuals becoming more cautious about preventive measures as the pandemic progresses [48]. Although the majority of X users fall within the young adult category (aged 18‐29 years), there are still a considerable number of older X users in the United States, as revealed by a survey conducted in 2023 [49]. Since this study calculated correlations for the entire period of data collection, these changes in opinion might not have been captured well. Throughout the study period, there have been several changes in mask-wearing rules, compliance, and when masks are mandated in some areas, which also affects how people responded and their opinion towards mask-wearing [50]. New information about mask-wearing released by the authority can also result in changes in public attitude toward mask-wearing [51], such as when people initially support mask-wearing and then change their opinion, or if they are against masking in the beginning and change to accepting masks in the later stage of the pandemic. This was also supported by results of the study, where a change in the sentiment is observed in the 3 major cities in the United States [15].

In Japan, the number of people aged older than 65 years shows a positive correlation with the number of tweets expressing their stance against masking, in line with a previous study in Hiroshima, Japan [52]. Based on existing studies summarizing responses in 27 countries, including Japan, people over 60 did not really comply with wearing masks outside of their home [53], which is also reflected in the results of this study [54]. The overall results of the correlation calculated are, however, quite small. Such a result indicated that there could be more confounding factors influencing the results. Below, we discuss some potential factors suggested in previous literature.

Health policy differences in the states within the United States might be one of the possible factors affecting the mask-wearing behavior reflected in social media. There had been observable differences, such as physical distancing rules across states and the relation of the measure to racial and socioeconomic differences in the area [55]. There was also an existing study documenting mask-wearing policy across the states and its enforcement [56]. The result indicates that overall, the mask is not strongly enforced in the states. The resistance to mask-wearing in the United States could also be viewed through cultural aspects of people living in the country [57]. A study focusing on the cultural and political aspects of mask-wearing suggested that future attempts at mask-wearing policy need to consider the cultural meaning of masks to the public [58].

A difference in response to policies was observed in Japan, with results from a study showing the high trust and compliance in government policies during COVID-19 [59]. An internet survey showed that some habits of the Japanese, such as wearing masks prior to the pandemic, were also related to their responses to the preventive measures for COVID-19 [60]. These policies and behavioral insights might serve as additional variables to further explain the observed web-based behavior in X. The insights can be used in handling information dissemination in social media, for example, by the government in countries to design the most effective way to communicate with the public on social media.

#### Interpreting Conflicting Correlations: Mobility, Social Media, and Educational Shifts

Most of our findings aligned with previous research. However, unexpectedly, individuals with a bachelor's degree or higher also showed a positive correlation with opposition to the mask mandate, which contradicts earlier studies [48]. One possible cause for the inconsistency was the difference in the demographics between people using X and the census data used for the correlation calculation. In the United States, the percentage of X users with at least a bachelor’s degree is higher than that of the general public, which is also observed in the income levels [61]. In Japan, based on a survey conducted by the Ministry of Internal Affairs and Communications, about 80% of people in their 20s were X users. The penetration rate is the highest in the younger population, but there were also users in their 60s (18.9%) [62]. According to a study conducted using X data, even within the social media users’ demographics, there was a bias due to the choice to participate in a certain topic for social media discussion [63]. This disparity might affect the correlation results with tweet percentages, as the demographics being calculated are not X-specific demographics but public demographics based on census and surveys.

Another possible cause of the inconsistency in results may also have been caused by the mobility of people throughout the COVID-19 period in Japan. The tweet data were collected over a 3-year period, while the education level was obtained from the 2020 census. A previous study mentioned that before the pandemic, people were centralized in the Tokyo metropolitan area, a pattern disrupted during COVID-19 [64]. The changes in migration rates were also influenced by the emergence of remote work during the pandemic, prompting younger people to move to rural areas.

The increase in tweets mentioning “no-mask,” as shown in the study by Suzuki et al [39], might also contribute to the conflicting correlation results, particularly in the latter half of 2022. Another reason might be caused by changes in the education style during the early period of COVID-19 to adapt to the changing policy. The changes to web-based education proved to be overwhelming for students, with their self-reported mental health status being worsened [65]. The anxiety and depression experienced during COVID-19 also increased students’ social media usage [66]. This might be reflected in opinions about COVID-19 and its measures, such as mask-wearing, where similar cases were observed in studies about disruptions caused by COVID-19 in social media [42].

Our findings indicated that mask-wearing behavior was observed in social media discourse in both the United States and Japan. The map showing the area with a high percentage of yes and no to mask-wearing can be considered a guideline in how to communicate or release guidelines in the event of a pandemic, with more attention to the area with a high percentage of “no” in mask-wearing. Based on the number of tweets, we may also launch more information on social media to reach more people in the area. The correlation results show some significant correlations observed with population data of the states and prefectures, with some of them consistent with existing findings. These results suggest that the social media data has the potential to aid in research, with some caution due to the limited nature of the geo-tagged data in social media. Focusing on other aspects of analysis, such as other potential factors in correlation analysis or additional data, can be a future research direction to improve the results.

The Health Belief Model was believed to be useful for understanding and investigating the participation in COVID-19 preventive measures in the United States [67]. A study using the Understanding America Survey showed that the Health Belief Model results were consistent with existing studies, with mask-wearing behavior positively endorsed as one of the preventive measures. A study comparing public perceptions in different countries during the pandemic, Japan and the United States included, also showed that higher perceived effectiveness was one of the factors affecting how the public responded to preventive behavior, such as mask-wearing or handwashing [68]. To make the public more accepting of preventive measures or policies, the public needs to trust the effectiveness of the act. The role of publishing and spreading the effectiveness can be entrusted to social media, with emphasis in areas with higher percentages in the stance of not wearing masks.

### Limitations

This study focuses solely on the English and Japanese languages for analyzing short and informal text, specifically tweet posts. In the second stage, we encountered numerous ambiguous and unclear tweets regarding attitude. Analyzing such stages proved challenging due to the short text and ambiguity of tweet content, which was often insufficient for accurately determining attitudes. Therefore, we chose to exclude a neutral option, limiting choices to either against or not.

Although X data might be subject to sampling bias due to its limited user base, the data were proven useful for infodemiology research, especially in identifying and monitoring public discussion topics during the pandemic [69,70]. Population-wise, our sample is restricted to individuals who voluntarily provide location information in their tweets. Despite the limitation of such data, it reflects the opinion of the users to a degree, as reported in an existing study that showed that geotagged tweets were able to display public opinion of people in the United States toward COVID-19 vaccinations [71]. Disparities in tweet counts across states/prefectures may influence the final percentage results displayed in the figures, which are based on the entire 3 years of data. We calculated the percentage based on the tweets observed in each state as one of the measures to address the potential problems due to the location data. The number of tweets in each state varies, so we avoid general bias caused by comparing the tweets based on their count, but based on the percentage of the classified tweets. Due to the data collection period and collective analysis after, the resulting figure, especially in the form of map visualization, may not sufficiently capture early COVID-19 period changes and post-pandemic trends shown in the time series plot.

A previous study identified around 8.4% of tweets as bots from around 200,000 accounts collected in the early period of the pandemic, March to June 2020 [72]. Another study mentioned that bots focused more on political content in the COVID-19 pandemic discourse compared with health content, such as actions taken by the public during the pandemic [73]. The bots mostly discussed information related to COVID-19 or pandemic statistics. In this study, we focused on mitigating bots’ influence, such as artificial amplification by removing duplicate tweets, one of which often appeared in posts by bot accounts. This action ensured that no single automated message could statistically dominate our social media dataset or skew our demographic correlations. To verify this approach, we conducted a manual audit in the annotated dataset, which was sampled randomly from the entire tweets data. We did not find apparent bot accounts during the check in our sample. Given this minimal presence, we conclude that the presence of bots does not significantly skew the overall stance analysis or correlations in our study.

The limited number of “not wearing” mask annotations constrained the number of predicted instances in the rest of the tweets. Consequently, the final predicted percentages for “not wearing” masks were also very small, only 0.07% for English tweets and 2.33% for Japanese tweets. This problem, caused by a small number of tweets annotated as “not wearing mask” was also further added by a surge in tweets mentioning “no-mask” after June 2022 [39]. This surge, as documented by Suzuki et al [39], may contribute to the disparity from previous studies and introduce bias into the correlation calculations, requiring people to interpret the results cautiously. To address these challenges, future improvements in classification and additional annotations are necessary for enhanced accuracy.

The initial score of the classification results obtained was not satisfactory. The *F*_1_-score for stance against masking is 0.61 for English and 0.69 for Japanese. It was even smaller for the mask-wearing stage, with 0.31 for English and 0.40 for Japanese. While it is higher than the majority baseline, it is still not performing as well for the classification. There is a slight increase in the *F*_1_-score after adding pseudo-labeled data, as observed in the stance and mask-wearing stage. However, the scores were still not as high, indicating that the topic to be classified is indeed challenging, as both human annotators and classifiers struggle to achieve a high score in classifying stance against masking and mask-wearing information of the users.

Regarding the mask-wearing stage, the number of tweets annotated as “not-wearing” masks is very small in the annotation phase (10 for English and 11 for Japanese). This small number affects the classification because when we split the data, we were working with an even smaller number of tweets for training, validation, and testing. This makes it even harder to classify the “not-wearing-mask” tweets, resulting in a low *F*_1_-score in the testing phase. This issue is further highlighted in the classification of the remaining tweets, with only 0.07% predicted as “not-wearing” masks in English and 2.33% in Japanese out of all the predicted tweets. The low number of tweets predicted as “not-wearing” masks is also apparent in Figure 3, especially in the United States case. This limitation warrants caution in interpreting our results, especially since the annotation target was particularly challenging.

Due to the policy of X, we are unable to obtain the demographics of its users; thus, we considered using the demographics of the general population in the country. However, there are differences between the demographics of X users and the general population [61,62] which might affect the results of the correlation calculated in this study.

### Conclusions

Our findings revealed a clear shift in public opinions at several key moments, often coinciding with major policy changes or public events, such as mask mandates and the Olympics, observed from 1,102,876 English tweets and 560,873 Japanese tweets from the United States and Japan between early 2020 and late 2022, covering the entire duration of the COVID-19 pandemic. Based on the correlation observed in the social media data, some variables are significantly correlated with the number of tweets, such as education levels, the population of the elderly, and household income. Some of our findings align with previous research across major social media platforms, suggesting that public opinions reflected in social media data are often interconnected. However, divisions based on education level, household income, and generational differences also indicate that social media interactions may be inherently biased, partly due to recommendation algorithms that encourage clicks and foster echo chambers.

## Abbreviations

BERT: Bidirectional Encoder Representations from Transformers
CDC: Centers for Disease Control and Prevention
SoE: state of emergency
